# Establishment of endolithic populations of extremophilic Cyanidiales (Rhodophyta)

**DOI:** 10.1186/1471-2148-6-78

**Published:** 2006-10-05

**Authors:** Hwan Su Yoon, Claudia Ciniglia, Min Wu, Josep M Comeron, Gabriele Pinto, Antonino Pollio, Debashish Bhattacharya

**Affiliations:** 1 Department of Biological Sciences and Roy J. Carver Center for Comparative Genomics, University of Iowa, Iowa City, Iowa 52242, US; 2 Dipartimento di Biologia vegetale, Università "Federico II", via Foria 223, 80139 Napoli, Italy

## Abstract

**Background:**

Cyanidiales are unicellular extremophilic red algae that inhabit acidic and high temperature sites around hot springs and have also adapted to life in endolithic and interlithic habitats. Comparative genomic analysis of *Cyanidioschyzon merolae *and *Galdieria sulphuraria *predicts that the latter may be more broadly distributed in extreme environments because its genome contains membrane transporters involved in the uptake of reduced carbon compounds that are absent from *C. merolae*. Analysis of an endolithic site in the Phlegrean Fields near Naples, Italy is consistent with this prediction showing this population to be comprised solely of the newly described lineage *Galdieria*-B and *C. merolae *to be limited to humid habitats. Here, we conducted an environmental PCR survey of another extreme environment in Tuscany, Italy and contrasted Cyanidiales population structure at endolithic and interlithic habitats in Naples and Tuscany.

**Results:**

We find a second *Galdieria *lineage (*Galdieria*-A) in endolithic and interlithic habitats in Tuscany but surprisingly *Cyanidium *was also present at these sites. The photoautotrophic *Cyanidium *apparently survives below the rock surface where sufficient light is available for photosynthesis. *C. merolae *is absent from all endolithic and interlithic sites in Tuscany. Population genetic analyses of a partial calmodulin gene fragment suggest a recent establishment or recurrent gene flow between populations in Tuscany, whereas the highly structured *Galdieria*-B population in Naples likely originated from 2–3 founder events. We find evidence of several recombination events across the calmodulin gene, potentially indicating the presence of sexual reproduction in the Tuscany populations.

**Conclusion:**

Our study provides important data regarding population structure in extreme endolithic environments and insights into how Cyanidiales may be established in and adapt to these hostile environments.

## Background

**Figure 1:**
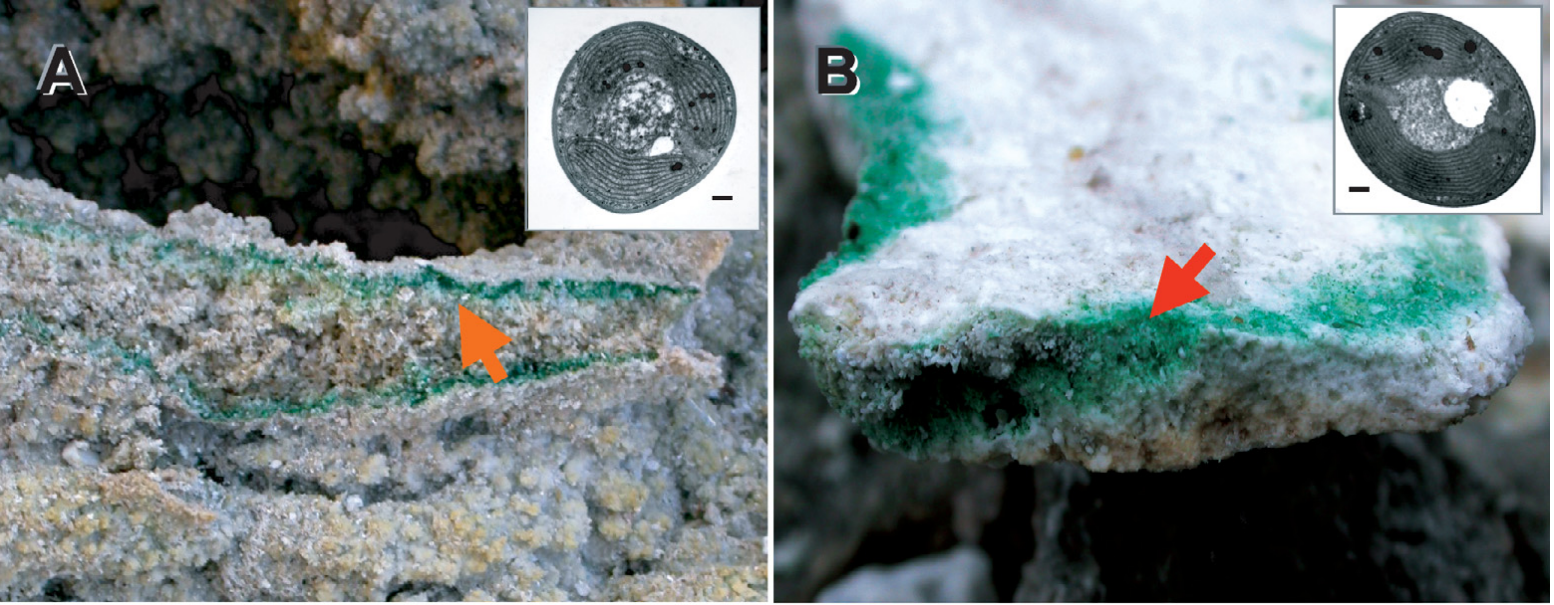
The endolithic Cyanidiales at Larderello in Tuscany (A) and in Pisciarelli, Naples, Italy (B). The arrows indicate the biomat of Cyanidiales that thrive inside of the rock at these sites. The TEM images show *Galdieria*-A (A) and *Galdieria*-B (B) from the endolithic sites. Scale bars = 1 μm.

The endolithic environment affords microorganisms protection from desiccation, rapid temperature variation, and UV radiation flux [1-3]. This microhabitat may, however, be extremely challenging, in particular for photosynthetic organisms because of limiting light (e.g., 1% of daylight) and nutrient conditions [1]. The Cyanidiales are unicellular red algae that thrive in endolithic habitats (see Fig. 1A, 1B) [1,4].

As eukaryotic extremophiles, Cyanidiales grow at high temperature (50° – 55°C) and in extremely acidic (pH 0.5 – 3.0) habitats around hot springs and/or acidic sulfur fumes [5-8]. Because of their simple morphology, only three genera (*Cyanidium, Cyanidioschyzon*, and *Galdieria*) and six species of Cyanidiales have historically been recognized [5,9-12]. However, we recently discovered a high biodiversity of Cyanidiales at sites in Pisciarelli in the Phlegrean Fields near Napoli, Italy that included three putative new genus/order-level lineages. The results of this study suggested the one of the new lineages, provisionally named *Galdieria*-B, was adapted to and dominated the endolithic habitat [6].

Among previously described Cyanidiales, members of the genus *Galdieria *are distributed among inter- and endolithic habitats in addition to diverse microhabitats including non-thermophilic sites [1,5,6,12-14]. This genus is heterotrophic and mixotrophic, can grow on more than 50 carbon sources, and tolerates a broad range of pH (0.5 – 6.0) and salinity (4 – 10%) [5,12]. The metabolic flexibility of *Galdieria *may explain why this genus can thrive in diverse environments in contrast to *Cyanidioschyzon merolae *which appears to be limited to humid habitats [6]. A recent comparative genomic analysis of these two species supports this view showing the presence of a large number of membrane transporters involved in the uptake of reduced carbon compounds in the *Galdieria sulphuraria *genome that are absent from *C. merolae *[15]. However there is no direct evidence from physiological studies in *C. merolae *to support these predictions based on genome analyses.

Here a culture-independent, environmental PCR survey approach was used to examine Cyanidiales species composition in nature. This method is a very efficient approach to facilitate the discovery of uncultivated cryptic species, particularly for unicellular microbes that may lack distinguishing morphological characters [6,16]. We conducted an environmental PCR survey from five inter- or endolithic populations in two geothermal areas at Larderello, a geologically active area in southern Tuscany, Italy (Fig. 1A). Using the ribulose 6-phosphate carboxylase oxygenase (*rbc*L) coding region as a marker, we determined species composition at these sites and compared these data to a typical endolithic population that was previously described from Pisciarelli (PBen site, Fig. 1B) [6]. In addition, we determined a partial sequence of the calmodulin (*CaM*) gene that includes 2 or 3 spliceosomal introns from multiple individuals to address Cyanidiales population structure. Phylogenetic analyses of *rbc*L sequences from the environmental samples revealed a clear pattern of genetic differentiation among the studied Cyanidiales with the *CaM *data revealing different species composition patterns in the endolithic microhabitats.

## Results

### *rbcL *phylogeny and species distribution of Cyanidiales

**Figure 2:**
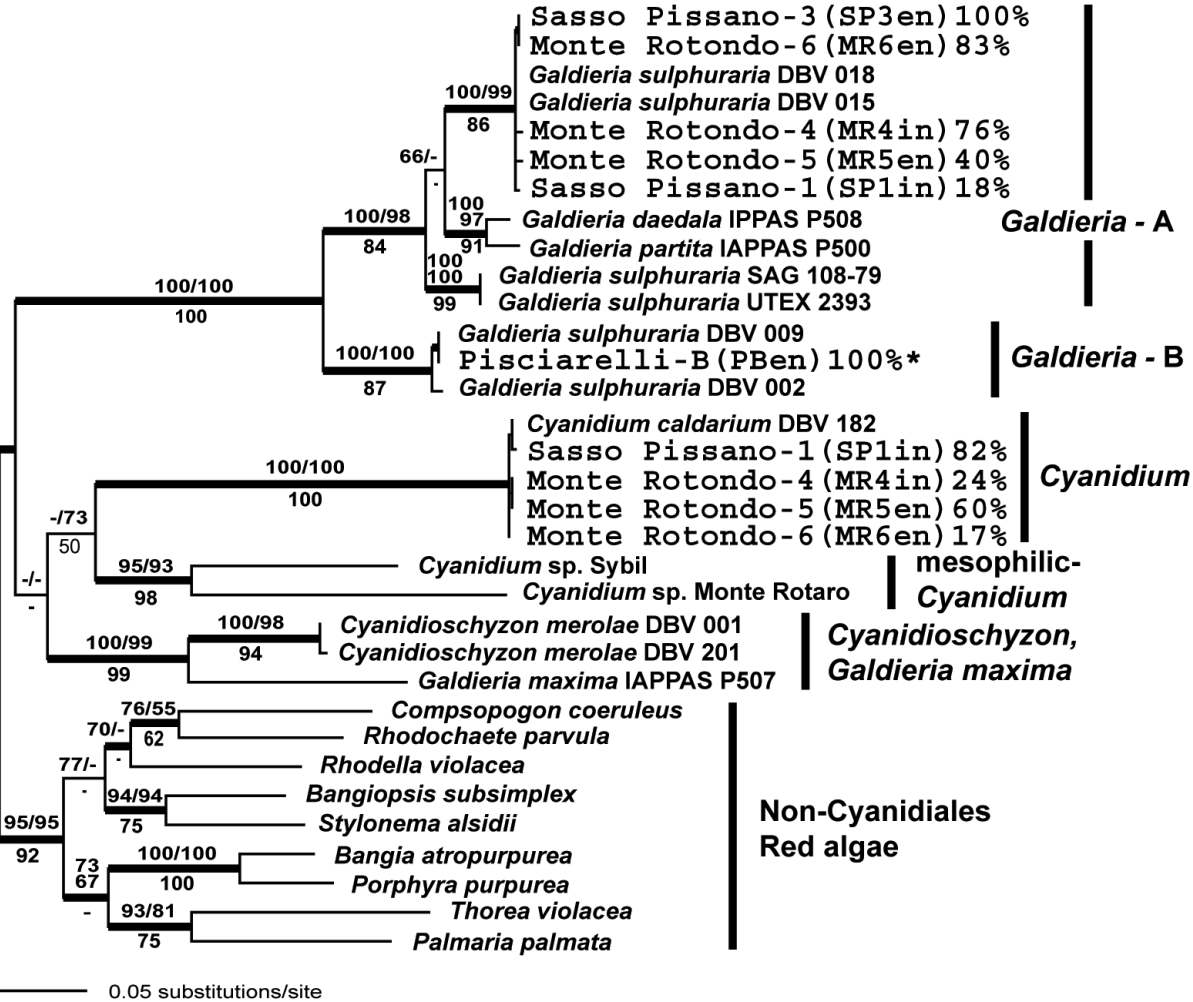
Phylogeny of the Cyanidiales inferred from a maximum likelihood (ML) analysis using the site-specific GTR evolutionary model and *rbc*L sequences. Results of the ML-all bootstrap analysis are shown above the branches on the left of the slash mark, whereas the results of the bootstrap analysis of ML-1^st ^+ 2^nd ^data set are shown on the right of the slash mark. The results of the protein maximum likelihood bootstrap analysis using the JTT + Γ model are shown below the branches. Only bootstrap values ≥ 50% are shown. The thick nodes represent ≥ 95% Bayesian posterior probability for clades using the site-specific GTR model. The sequences arising from a previous study [6] are marked with asterisks. The percentage values shown for each sampling site indicate the proportion of Cyanidiales members that were recovered in the different PCR-based environmental libraries. About 20 clones were sampled from each site (for the actual number, see Table 1).

The ML trees of *rbc*L sequences were inferred from three data sets of 1215 nt (all three codon positions, ML-all), 810 nt (excluding 3^rd ^codon positions, ML-1^st ^+ 2^nd^) and 405 aa (ML-protein) from 9 new environmental samples of Cyanidiales that included 90 representatives. The trees inferred from the latter two analyses share a very similar topology with the ML-all tree (Fig. 2), except for the monophyletic clade composed of *Cyanidioschyzon merolae *and *Galdieria maxima *that is positioned with the *Galdieria*-A + -B lineage in the ML-protein tree. However this clade was poorly supported in the bootstrap analyses (i.e., < 50%). The *rbc*L phylogeny is similar to a 3-gene tree found in our previous study [6]. The ML-all tree shows strong support for the monophyly of the Cyanidiales, with these taxa forming a sister group to the rest of the red algae (ML-all bootstrap = 95%, ML-1 + 2^nd ^= 95%, ML-protein = 92%, and BPP = 1.0, Fig. 2). The internal topology of the Cyanidiales indicates a division into four lineages [6]; 1) *Galdieria *spp. (*Galdiera*-A and -B lineages, excluding *Galdieria maxima*), 2) *Cyanidium caldarium*, 3) mesophilic *Cyanidium *sp., and 4) *Cyanidioschyzon merolae *and *Galdieria maxima*. Each of these lineages was strongly supported in the bootstrap analysis, however their interrelationships are poorly resolved (Fig. 2). As apparent in the tree *G. maxima *is clearly separated from the other *Galdieria *species (e.g., *G. sulphuraria, G. daedala*, and *G. partita*) and shows a close relationship to *C. merolae*. This result is congruent with our previous study [6] and suggests that a taxonomic revision is required for the genus *Galdieria*.

We sequenced a total of 90 *rbc*L-encoding clones from five sites. All of these clones were positioned in the *Galdieria*-A and *Cyanidium *lineages. Members of the *Galdieria*-A lineage were found at Monte Rotondo-4 (MR4in) (13/17 clones), Monte Rotondo-5 (MR5en) (6/15 clones), Monte Rotondo-6 (MR6en) (15/18 clones), Sasso Pissano-1 (SP1in) (4/22 clones), and Sasso Pissano-3 (SP3en) (18/18 clones). All clones from the SP3 site were however restricted to the *Galdieria*-A type. We did not find any representatives of the *Galdieria*-B lineage from the Larderello populations. This is in contrast to Pisciarelli which included an endolithic habitat (i.e., PBen, 19/19 of *Galdieria*-B) [6]. It is noteworthy that members of *Cyanidium *were found in many Larderello populations (MR4in, 4/17; MR5en, 9/15; MR6en, 3/18; and SP1in, 18/22) but were absent from the PBen site [6].

### Population structure inferred from calmodulin intron sequences

The 18 *rbc*L sequences of *Galdieria*-A from the SP3en site were virtually identical except for 1–2 non-informative substitutions per insert among the ca. 700 bp that were sampled. Similar to the SP3en site, the Pisciarelli-B (PBen) clones also consisted of virtually identical *rbc*L sequences of *Galdieria*-B [6]. To address the population structure at these sites, we chose to sample a more rapidly evolving nuclear sequence. Therefore, we determined a partial sequence of the *CaM *gene from DNA clones isolated from the SP3en and PBen sites. Twenty sequences from SP3en and 15 sequences from Pisciarelli-B were determined. Because SP3en showed a relatively high divergence rate within the *CaM *spliceosomal introns, we determined more sequences from MR4in (2 seq.), MR5en (14 seq.), and MR6en (15 seq.) to address this finding at the Larderello sites. In addition, a homologous region of the *CaM *gene from *Galdieria sulphuraria *DBV-009 and UTEX 2393 strains was also determined as reference. *Cyanidioschyzon merolae*, which lacks introns in the *CaM *gene (CMK219C from the *Cyanidioschyzon merolae *Genome Project), was used to determine the sites of intron insertion.

**Figure 3:**
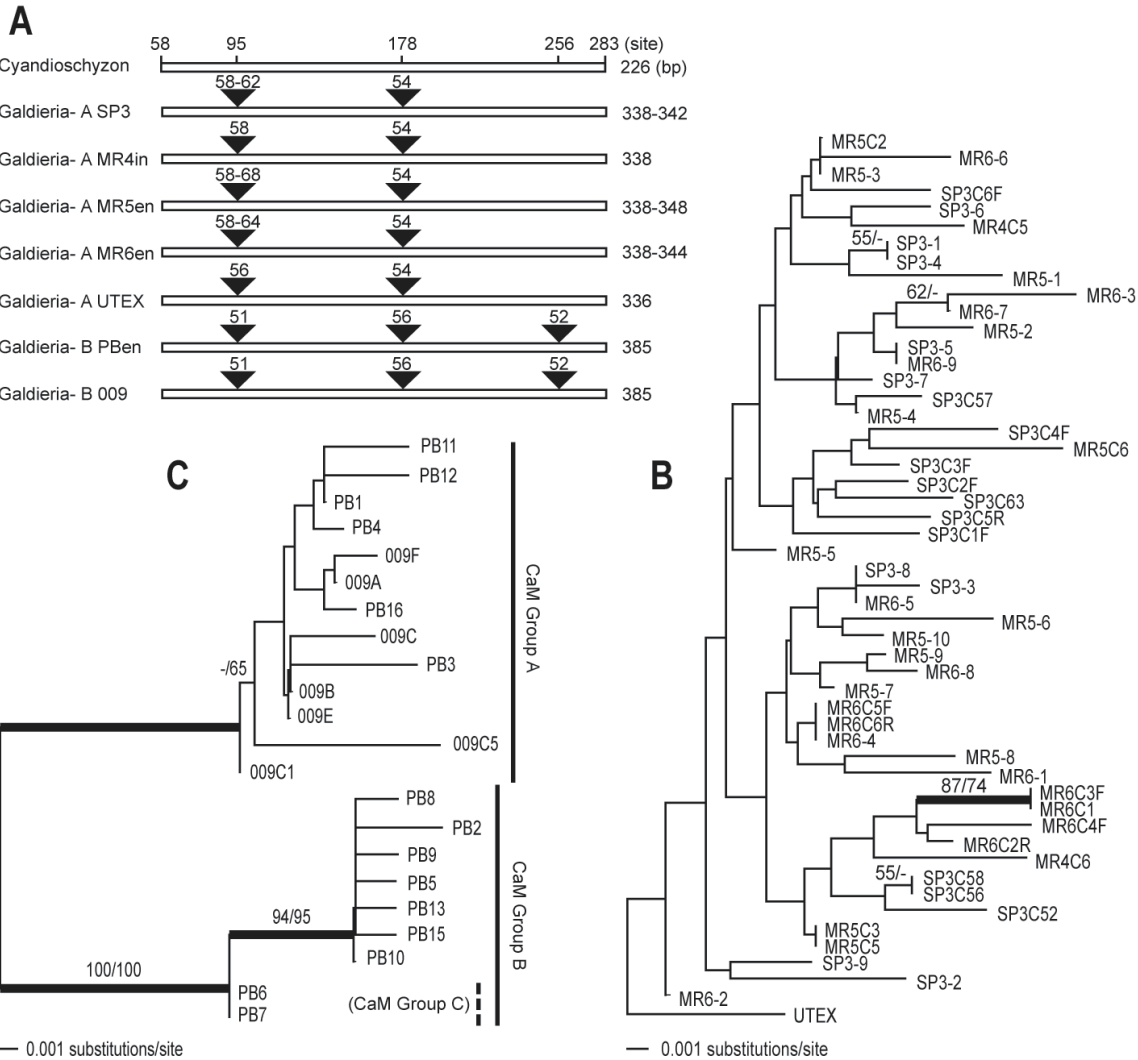
The intron positions in the *CaM *gene of Cyanidiales (A). Phylogeny of the Cyanidiales from Larderello (B) and Pisciarelli (C) inferred from a minimum evolution (ME) analyses of partial *CaM *gene sequences (including introns) using the HKY85 model. The bootstrap values shown above the branches are from ME (left of the slash mark) and MP (right of the slash mark) analyses. Only bootstrap values ≥ 50% are shown. The thick nodes represent ≥ 95% Bayesian posterior probability for clades using the GTR + I + Γ model.

The partial *CaM *coding region (nucleotides 58 – 283^rd ^position in *C. merolae*) showed size variation (338 – 342 nt in length) in the SP-3 population (*Galdieria*-A). Two introns were found following the 95^th ^and 178^th ^nucleotide positions in the alignment and were of size 58 – 62 nt and 54 nt, respectively (Fig 3A). There were guanine and thymine (GT) repeats in the first intron (4x – GT, 8 clones; 5x – GT, 5 clones; 6x – GT, 7 clones) that gave rise to the size variation. In contrast, the MR populations (MR4in, MR5en, and MR6en) showed a 4 – 9x GT repeat and the *G. sulphuraria *UTEX 2393 gene contained 3x- repeats in the first intron. There was no size variation (385 nt) in the *CaM *gene from the PBen site (*Galdieria*-B). The second intron (52 nt) was found in addition to the first (51 nt) and the third following site 178^th ^(56 nt) in the PBen population. Although two single-species populations share intron sites (95^th ^and 178^th^), the intron sequences are too divergent between the two populations to be aligned. Therefore, we analyzed the PBen data separately using the *CaM *gene plus the intron sequences. Although these data showed much less variation most of the polymorphic sites are likely to be real changes rather than due to *Taq *polymerase mis-incorporation or sequencing errors due to the distribution of many as shared or silent changes. Analysis of the distribution of polymorphic sites showed that of 37 columns with changes in the alignment, 18, or 49% were comprised of changes that were shared among different clones (i.e., individuals), 19% were in third codon positions, and of the other 12 unique changes, 9 were in the relatively divergent intron regions.

The ME tree using the *CaM *sequences shows a highly diverse branching pattern for the SP3en population which is comprised of a single species (*Galdieria*-A). Most nodes are weakly supported. When we included the three Larderello populations (MR4in, MR5en, and MR6en), the representatives of these sites were intermixed with the SP3en taxa, however without strong bootstrap support (Fig. 3B). In the ME analysis of the PBen population (*Galdieria*-B) that is shown in Figure 3C there is a clear separation of the sequences into two (possibly three) lineages. Six clones of the *CaM *gene form a well-supported monophyletic group (PB-CaM Group A) with *G. sulphuraria *DBV-009 (ME = 100%, MP = 100%, BPP = 1.0). Two representatives (PB6, PB7; tentatively PB-CaM Group C) of PB-CaM Group B are relatively distantly related from the rest of this clade (ME = 94%, MP = 95%, BPP = 1.0). The remainder of the clones form another distinct clade named PB-CaM Group B.

### Population differentiation

Congruent with the phylogenetic studies, there is no fixed nucleotide difference between sequences from the SP3en and MRs (collective term for MR4in, MR5en, MR6en) populations. Out of the 38 observed mutations in the SP3en/MRs populations, 11 are shared between populations (mutations segregating within both populations) and 27 are present in only one population. To investigate whether the SP3en and MRs sites constitute genetically differentiated populations, we applied population differentiation tests that take into account the frequency of mutations within and between populations (Table 1). The results reveal detectable differentiation between the SP3en and MRs populations and hence, and despite the similarity, we can rule out a single panmictic population constituted by the SP3en and MRs sites.

**Table 1:** Proportions of Cyanidiales Taxa at the Different Collection Sites in Italy

**Taxa**	**Population**	**Abbreviation**	***rbc*L**	**Calmodulin**
**Larderello, Tuscany**				
*Galdieria*-A	UTEX 2393	UTEX	AF233069	**DQ916754**
	Monte Rotondo-4	MR4in	**DQ916745 **13/17	**DQ916775–776**
	Monte Rotondo-5	MR5en	**DQ916746 **6/15	**DQ916777–790**
	Monte Rotondo-6	MR6en	**DQ916747 **15/18	**DQ916791–805**
	Sasso Pissano-1	SP1in	**DQ916748 **4/22	-
	Sasso Pissano-3	SP3en	**DQ916749 **18/18	**DQ916755–774**
*Cyanidium caldarium*	Monte Rotondo-4	MR4in	**DQ916750 **4/17	-
	Monte Rotondo-5	MR5en	**DQ916751 **9/15	-
	Monte Rotondo-6	MR6en	**DQ916752 **3/18	-
	Sasso Pissano-1	SP1in	**DQ916753 **18/22	-
**Pisciarelli, Naples**				
*Galdieria*-B	DBV 009 VTNE	009	AY119768	**DQ916806–812**
	Pisciarelli-B	PBen	AY541314–316 19/19	**DQ916813–827**

**Column headings**: The taxon and site names and GenBank accession numbers for the *rbc*L and calmodulin genes that were isolated in this study are shown. The ratios in the *rbc*L column are the actual number of clones of the taxon over the total number of PCR clones that were sequenced from each environmental library.

**Figure 4:**
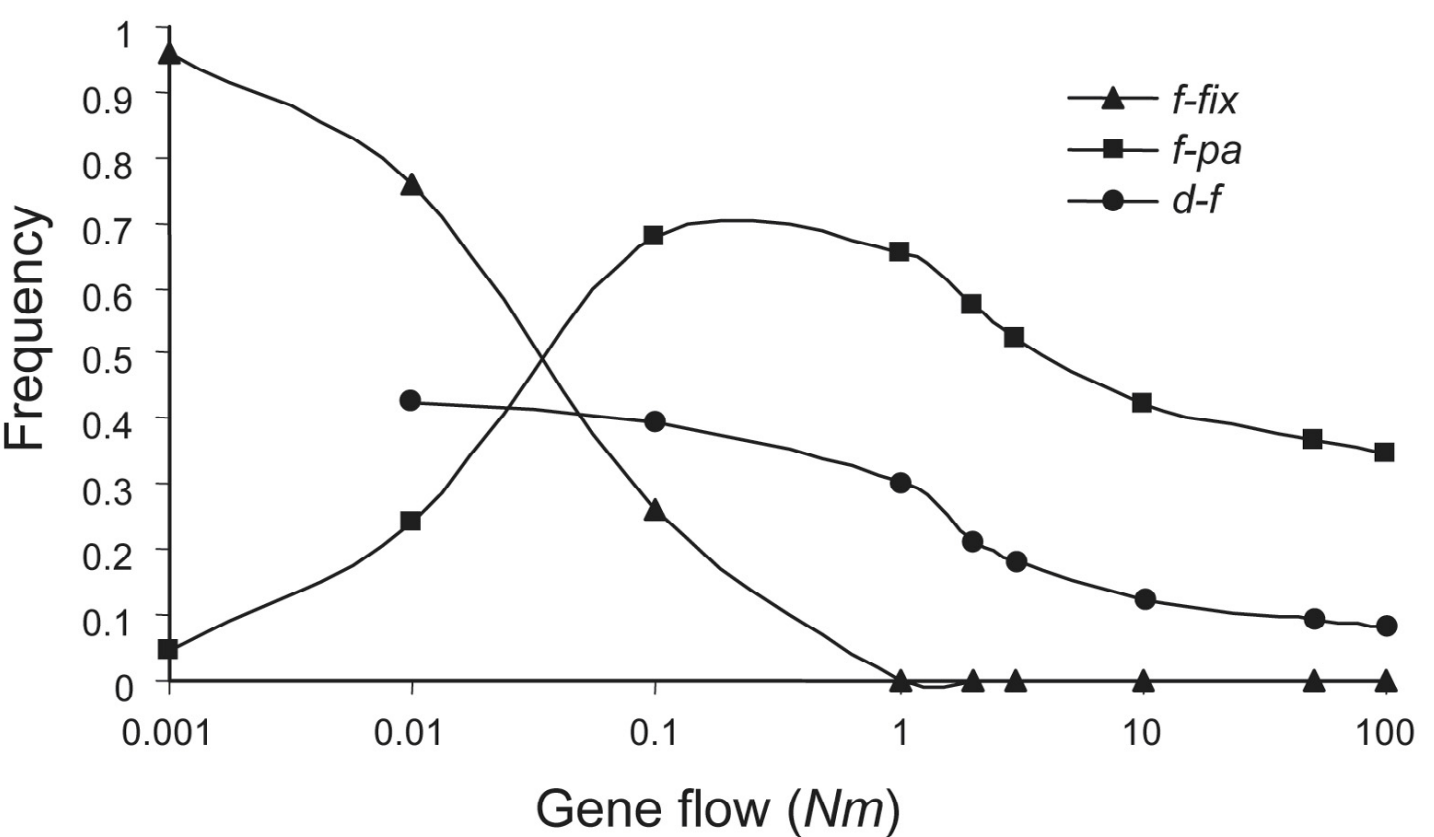
Expected influence of gene flow on genetic differentiation between two populations. Gene flow is measured as *Nm *(the product between the effective population size, *N*, and the migration rate, *m*) and genetic differentiation is investigated by means of the frequency of fixed mutations between the populations (*f-fix*), the frequency of polymorphisms present in a single population (*f-pa*), and the difference in frequency between mutations shared between both populations (*d-f*). The results are based on coalescent simulations under different conditions of gene flow and conditional to the number of observed mutations and the number of chromosomes. In our analyses comparing SP3en and MRs sites, *f-fix *is zero, the observed *f-pa *is 0.71, and the observed *d-f *is 0.095, suggesting *Nm *> 1.

This weak, albeit detectable, differentiation may indicate extensive gene flow between SP3en and MRs or a very recent establishment of two isolated populations, where shared mutations would represent ancestral polymorphism. Both scenarios predict that shared mutations should be at similar frequencies in both populations. Indeed, this pattern is observed between SP3en and MRs populations (r^2 ^= 0.35, P = 0.0001), with an average difference in the frequency of only 0.095. The amount of gene flow required to completely eliminate fixed differences between two long-term isolated populations and cause mutations to segregate at similar frequencies is very high (Nm > 1, where N is the average effective population size and m is the migration rate; see Fig. 4). On the other hand, extensive gene flow is unlikely to be associated with ~71% (27 out of 38) of mutations being present in only one population, tentatively suggesting that a recent establishment of two isolated populations is the most likely scenario.

## Discussions and conclusions

The Cyanidiales provide an untapped resource for understanding adaptations at the genomic level to life in extreme environments with one complete genome sequence already available (*Cyanidioschyzon merolae *10D) [15,17] and another that is nearly finished (*Galdieria sulphuraria *074G) [18]. The Cyanidiales are unique in their ability as eukaryotes to thrive under extreme conditions at hot springs (temperatures between 50° – 55°C, pH between 0.5 – 3.0) [5-8,19]. These single-celled, photosynthetic red algae also have the ability to grow under low light conditions in endolithic habitats (see Fig. 1) [1,4,6]. It is rare for eukaryotes to live beneath the rock surface, although endolithic prokaryotic communities have often been found in extreme conditions (e.g., deserts, hot spring, and arctic regions) [3,4]. Endolithic Cyanidiales were first reported from Pisciarelli and La Solfatara near Naples, Italy [1]. These authors assumed, based on gross morphology (i.e., cell size from SEM images, see Fig. 3 in Gross *et al*. [1]) that the endolithic Cyandiales were species of *Cyanidium *and *Galdieria*. However, traditional taxonomic characters are not very useful in identifying morphologically simple, coccoid Cyanidiales cells [5,6]. We recently surveyed environmental samples from Pisciarelli and found a new lineage termed *Galderia*-B that dominates the endolithic habitat [6]. It was postulated that *Galdieria*-B had adapted to this relatively dry environment. Here we tested whether *Galdieria*-B also dominates the endolithic habitat at another site (Larderello, Tuscany, Italy) and contrasted the population structure in Pisciarelli and Larderello.

### *Galdieria*-A and *Cyanidum *exist in endolithic habitats

The *rbc*L tree (Fig. 2) clearly resolves the species composition at five interlithic (MR4in and SP1in) and endolithic (MR5en, MR6en, and SP3en) sites. Four sites (except SP3en) were composed of two species, *Galdieria*-A and *Cyanidium caldarium*. The SP3en site consisted of a single species (*Galdieria*-A). Individuals of *Galdieria*-A were more abundant in the endolithic populations (100% SP3en, 83% MR6en), however this species was also abundant at an interlithic site (76% at MR4in). *C. caldarium *was distributed in interlithic (82% SP1in, 24% MR4in) and endolithic (60% MR5en, 17% MR6en) habitats. It is surprising, despite our low sample size, that we did not identify other Cyanidiales species (i.e., *Cyanidioschyzon merolae, Galdieria*-B, and *G. maxima*) at the Larderello sites. *Galdieria*-B was of particular interest because it was rich in endolithic (100% PBen) and interlithic (40% P-C) sites at Pisciarelli [6]. These data suggest that several genetically divergent Cyanidiales taxa have likely adapted to flourish in the endolithic environment.

In contrast to sites around fumaroles or in hot acid springs, the endolithic environment appears to offer a relatively stable habitat. There is an absence of rapid temperature variation resulting from hot sulphur fumes, UV radiation flux, and desiccation (i.e., the rock provides moisture and protection from desiccation [1-3]. However, endolithic sites are limiting with respect to light – less then 1% of daylight is available for photosynthesis [1]. It may, therefore, be surprising that the Cyanidiales are able to thrive in endolithic environments. *Galdieria *has however been reported to be heterotrophic and mixotrophic and able to grow on more than 50 carbon sources [5,12,20]. *Galdieria *takes up a broad range of sugars and sugar alcohols using a variety of membrane transporters [20] that have been identified in the *G. sulphuraria *genome [15]. Gross *et al*. [1] demonstrated heterotrophic growth of *G. sulphuraria *using as nutrients an acid extract of autotrophically grown *Galdieria *cells. Therefore, it is possible that *Galdieria *grows photoautotrophically at the top of the biomat directly beneath the rock surface, whereas these cells grow heterotrophically in deeper regions of the algal layer, surviving on organic materials provided by dead cells [1].

Because of the apparent inability of *Cyanidium caldarium *to grow heterotrophically, it has not previously been regarded as an endolithic species [6,15]. However, our study provides evidence that *C. caldarium *can also thrive in endolithic sites. It is still unknown whether *C. caldarium *encodes the membrane transporters identified in the *Galdieria *genome, although this species has characters such as a cell wall, a central vacuole, and the osmolyte floridoside (as in *Galdieria*) [12,15] that are critical for heterotrophic growth. It is also possible that *C. caldarium *thrives without heterotrophy just below the rock surface where adequate light is available for photosynthesis [1,15]. These hypotheses need to be tested using controlled environmental conditions to assess their relevance.

Recently, another endolithic population was reported from Norris Geyser basin of Yellowstone National Park, USA [4]. These authors found four ribosomal (r) RNA sequences that were "closely related *Cyanidium *species" without providing species identifications. We included these sequences in phylogenetic trees (results not shown) and identified them as originating from *G. suphularia *(*Galdieria*-A; AY911434, AY911459), *G. maxima *(AY911496), and *C. merolae *(AY911454). The most intriguing observation is of *C. merolae *from an endolithic site, which contrasts to the finding of our study at Pisciarelli and Tuscany. It is generally thought *C. merolae *would not survive in the endolithic environment because it lacks a cell wall and a vacuole which are crucial for maintaining and regulating turgor pressure and the osmotic potential [5,12,15]. In addition, the genome of this alga does not contain many membrane transporter genes which appear to be critical for heterotrophic growth; i.e., it encodes only one monosaccharide transporter [15,17]. However, the genome of *C. merolae *does encode all the metabolic enzymes required for heterotrophic growth (i.e., GlcMan and Gal; for details see reference [15]). These genomic features are generally consistent with (but do not prove) the idea that the ecophysiological characters of *C. merolae *make it an obligatory photoautotroph that is limited to humid microhabitats such as in Pisciarelli, Naples, Italy [6]. Consistent with the predicted dominance of *C. merolae *in humid environments, Ferris et al [16], in a 18S rDNA-based environmental survey in Nymph Creek were able to recover only this taxon out of 162 clones that were isolated from this acidic stream. Nymph Creek is near Norris Geyser Basin in Yellowstone National Park. Despite these promising data, it is clear that understanding the true limits to the distribution of *C. merolae *in nature will require future sampling of many other sites.

### Establishment of endolithic Cyanidiales populations

How Cyanidiales colonize an endolithic habitat remains unknown. Gross *et al*. [1] suggested two possible mechanisms: 1) inoculation through cracks in the upper silica layer, and 2) the formation of the upper rock layer after Cyanidiales had colonized the rock surface, although it is unknown under which time scale the latter process may occur. If the endolithic sites were colonized by a single or a few cells via inoculation, these regions would potentially show low sequence diversity reflecting the chance entry of the founder cells. If the populations originated via covering of the rock surface, the sequence diversity would potentially be higher such as at non-endolithic (i.e., interlithic) sites. Selection for desiccation tolerance could however cause a selective sweep that would result in a strong reduction of genetic diversity after the origin of a covering rock layer. Upcoming multi-locus studies will allow us to discriminate between these two scenarios based on the fact that strongly advantageous mutations will influence polymorphism at particular genomic regions or chromosomes whereas recent colonization is expected to have genome-wide effects on polymorphism.

Because the *CaM *intron regions from *Galdieria *at the Larderello (*Galdieria*-A; SP3en, MR4in, MR5en, MR6en) and Pisciarelli (*Galdieria*-B; PBen) endolithic sites could not be reliably aligned these data sets were analyzed separately. The tree of the Larderello sequences shown in Figure 3B shows a paraphyletic radiation of *Galdieria*-A into the four sites. However, most nodes in this tree are weakly supported and there appears to be poor resolution of sequence interrelationships. This may indicate recurrent gene flow between the SP3en, MR4in, MR5en, and MR6en sites or extensive ancestral polymorphisms reflecting recent population establishment. Interestingly, *CaM *sequence diversity of the endolithic cells at the SP3en, MR5en and MR6en sites was similar to that of the interlithic MR4in site. In contrast, the *CaM *sequences from the endolithic Pisciarelli PBen site formed 2 distinct clades suggesting that two (or potentially three) independent inoculations of *Galdieria*-B occurred here. The significant differences between the topologies shown in Figures 3B and 3C are noteworthy and may provide important insights into how these endolithic populations were established.

A potential explanation for the difference in *CaM *gene diversity in Larderello versus Pisciarelli is that cells at the former site originated through the addition of a rock layer over an existing genetically diverse population. Gross *et al*. [1] provided preliminary support for this colonization method based on their finding that after the application of an artificial silica layer on *Galdieria *cells, these algae maintain a dark-green color for 4 weeks, even when the silica layer becomes 1 mm thick [1]. In contrast, the PBen population was likely colonized by inoculation of 2 – 3 founder cells. One of the founder cells at the PBen site is closely related to the DBV009 strain at the University of Naples culture collection that was isolated from a single colony.

### Recombination at intragenic level

The study of *CaM *sequences from the SP3en and MRs sites reveals an unexpected nucleotide pattern, with a minimum of 6 recombination events across the *CaM *region based on the conservative approach of investigating the presence of four gametic types [21] (see Methods for details). This observation is not compatible with a genetic system with the complete absence of genetic recombination (P < 0.0001). The study of the SP3en and MRs sites separately generates equivalent results, with a minimum of 4 and 5 recombination events, respectively (P < 0.0001 in both cases). These results are intriguing because they hint at the existence (present or in the recent past) of sexual reproduction in the Cyanidiales, a process that has never been observed in these algae either in culture or in the wild. Future population analyses across the genome will allow us better understand the evolutionary causes of this pattern.

## Methods

### Description of sampling sites

The geothermal sites for our collection in the Larderello region of Tuscany were Sasso Pisano and Monte Rotondo. Sasso Pisano contains several fumaroles and ''putizze'' which are vapours emanating from rocks, giving them a deep yellow hue. Pools are absent from this area and the surrounding soil is rubbly and hot with temperatures ranging from 25 – 45°C. Monte Rotondo has boraciferous fumaroles that flow from underneath the soil at a temperature ranging from 100 – 160°C. The biomats of Cyanidiales occur as thin layers on the rocks, between the crushed stones, and inside the rock layer (Fig. 1A).

To survey the endolithic Cyanidiales populations, we collected cells from the biomat at five different endolithic (or interlithic) sites (three in Monte Rotondo and two in Sasso Pissano). The Sasso Pisano-1 (SP1in) material was collected in a ravine of a crumbly wall between the detritus (this was an interlithic population). The temperature was ca. 29 – 31°C with a low pH (0.5 – 1.0). In the second sample (SP3en), the Cyanidiales biomat formed a thin layer inside a stone with the side containing the cells in contact with soil and never exposed to the sunlight. The pH at this site was low (1.0) and the temperature was ca. 30°C (this was an endolithic population). The Monte Rotondo-4 (MR4in) site had a population of Cyanidiales growing among trachytic and detritic rocks, on the hot soil (50°C, interlithic population). The Monte Rotondo-5 (MR5en) and Monte Rotondo-6 (MR6en) sites were endolithic in which the biomat grew inside the thin rock layers which were 0.5 cm in thickness at a depth of 0.3 cm. To collect Cyanidiales samples, we removed the surface rock using a hammer, and the cells were collected by scraping the thin algal layer into 50 mL Falcon tubes.

It is important to note that the endolithic condition at Sasso Pisano and Monte Rotondo was quite different from that of Pisciarelli [6]. The rock walls of the endolithic sites at Pisciarelli are strongly leached and appear hard, solid, and white due to the presence of alunite. The gases and vapors come from the surrounding fumaroles and pools with minor flow, and the temperature is always low (18 – 20°C). In contrast, at the Larderello geothermal endolithic sites, the rocks are more friable and crumbly, but neither opal nor alunite is present. Vapour arising from fissures heats these rocks.

### Environmental PCR

Genomic DNA was extracted from the environmental samples using the DNeasy Plant Mini Kit (Qiagen, Santa Clarita, CA, USA). Polymerase chain reactions (PCR) were done using the Cyanidiales-specific primers for the *rbc*L gene, which recognize conserved complementary sequences from members of all the different Cyanidiales lineages [6]. We are confident that there is no primer-bias or PCR-bias in our environmental PCR survey because we tested the efficacy of PCR-amplification using not only environmental materials but also DNA isolated from different cultured Cyanidales (for details, see [6]). The PCR products were purified (QIAquick PCR Purification kit, Qiagen) and cloned into the pGEM-T vector (Promega). A total of 90 clones were picked (up to 20 per site) and the inserts were sequenced using the BigDye™ Terminator Cycle Sequencing Kit (PE-Applied Biosystems, Norwalk, CT, USA), and an ABI-3100 at the Roy J. Carver Center for Comparative Genomics at the University of Iowa. One of the PCR primers (i.e., rbcL-90F) was used to provide preliminary sequence to identify the species of Cyanidiales from the 90 clones derived from the five separate environmental sites (sequence reads were approximately 700 nt in length). Thereafter, we completed the *rbc*L sequence (1215 nt) using additional sequencing primers. One colony was selected to represent each species found in the five environmental sites. These complete sequences were used for the phylogenetic analysis.

We chose two populations that consist of single species to address in detail their population structure (i.e., SP3en, this study; Pisciarelli-B, [6]). A partial fragment of the calmodulin gene (*CaM*) that occurs in single copy and contains 2–3 introns was used for this purpose. Four novel degenerate primers were designed to amplify the *CaM *gene based on available data from the *Cyanidioschyzon merolae *Genome Project [22] and the *Galdieria sulphuraria *Genome Project [23] (with the aid of A. Weber, Michigan State University): Calmo140F; 5'-GAA KCR GAR TTR MGA GAR ATG AT-3', Calmo160F; 5'-GAR ATG ATH GCV GAR GTW GA-3', Calmo510R; 5'-CTT CMT CBG TAA GYT TTT CTC C-3'; Calmo580R; 5'-ATC YGC TTC RCG AAT CAT TTC-3'. After amplification, the *CaM *fragment was cloned and sequenced as described above. We also included the *CaM *sequences from the *Galdieria sulphuraria *DBV-009 and UTEX 2393 strains as the positive control in these analyses.

### Phylogenetic analyses

All sequences were manually aligned using SeqPup [24] and are available upon request from D. B. For the *rbc*L gene, we included 24 representatives of the Cyanidiales that consist of 9 new sequences from Larderello (Tuscany), representing 90 individuals and 15 sequences from a previous study [6]. We used non-Cyanidiales red algae as the outgroup in this analysis. We prepared three data sets for the *rbc*L analysis to assess, and when necessary, to ameliorate the possible misleading effects of mutational saturation in the DNA sequences [5]. The first data set included all three codon positions (1215 nt), whereas the second excluded third codon positions (810 nt), and the third consisted of the translated to amino acid sequences (405 aa).

The maximum likelihood (ML) method was used to infer the phylogeny using the *rbc*L data. We used the site-specific, general time reversible model (ssGTR) [25] with estimations of nucleotide frequencies, the shape parameter of the gamma distribution with separate model parameter estimates for the 3 data partitions (i.e., 1^st^, and 2^nd^, and 3^rd ^codon positions) with the PAUP* computer program [26]. Ten heuristic searches with random-addition-sequence starting trees and tree bisection-reconnection (TBR) branch rearrangements were used to find the optimal ML tree.

To test the stability of monophyletic groups in the ML analyses, 1000 bootstrap replicates [27] were analyzed using the GTR + Γ + I model with the phyML program [28]. In the Bayesian inference of the DNA data (MrBayes V3.0b4) [29], we used the site-specific GTR + Γ (ssGTR) model. Metropolis-coupled Markov chain Monte Carlo (MCMCMC) from a random starting tree was initiated in the Bayesian inference and run for 1,000,000 generations. Trees were sampled each 200 cycles. Four chains were run simultaneously of which 3 were heated and one was cold, with the initial 400,000 cycles (2000 trees) being discarded as the "burn-in". A consensus tree was made with the remaining 3,000 phylogenies to determine the posterior probabilities (BPP) at the different nodes. For the analysis of data set of 1^st ^and 2^nd ^codon position, the same settings were implemented in the ML and Bayesian inference as described above except for the use of a 2-partition evolutionary model (i.e., 1^st^, and 2^nd ^codon positions).

For the protein rbcL data, a ML tree was inferred using "proml" (PHYLIP V3.6) [30] and the JTT + Γ evolutionary model with 10 random-sequence additions and global rearrangements. Bootstrap analysis was done with these data using the WAG + Γ + I model (1000 replicates) using phyML as described above. Bayesian inference of the protein data was done using the WAG + Γ + I model. For the *CaM *data set (340 bp), we used the minimum evolution (ME) method with HKY85 distances. Bootstrap analysis (1000 replicates) was conducted with ME and MP methods. The GTR + Γ + I model was used in the Bayesian inference of the *CaM *data.

### Population genetic analyses

To investigate population differentiation, we estimated several population statistics (Kst, Kst*, Zn, Zn*, and Snn) [31,32]. These statistics take into account, to a different degree, mutations that fully differentiate populations (i.e., fixed mutations), the possible difference in frequency of mutations that are shared between populations (i.e., shared polymorphisms), and the presence of segregating mutations in only one population (i.e., particular polymorphisms). The application of population genetic models, with different degrees of isolation and migration, to evaluate the most likely scenario is not appropriate in this case because both SP3en and MRs populations exhibit a strong departure from equilibrium predictions, with an excess of low-frequency variants (data not shown). The probability of observing population differentiation was obtained based on a permutation test with 1000 replicates.

To investigate the presence of intragenic recombination along the *CaM *sequence, we estimated the minimum number of recombination events (Rm) following Hudson and Kaplan [21]. Rm is based on the presence of four gametic combinations and underestimates the actual number of recombination events across a given sequence. Note also that Rm is not a population parameter for the rate of recombination hence it is independent of population genetics assumptions. A possible caveat to the use of Rm as a conservative measure for the presence of recombination would be a high incidence of recurrent mutations at a single site (i.e., sites with more than 2 variants) that could generate pairs of sites with four gametic combinations in the absence of recombination. We can, however, rule out this possibility because our SP3en/MRs-CaM data reveal that there is no site with more than 2 variants. The probability of observing Rm under conditions with no recombination was obtained using 10,000 coalescent simulations of the neutral process (a conservative approach when there is an excess of low-frequency variants), conditional to the observed number of polymorphisms and number of chromosomes under study [33].

## Authors' contributions

CC, AP, and GP collected Cyanidiales samples. CC, HSY, and MW did all the molecular work. JMC conducted the population genetic analyses and wrote this section of the manuscript. HSY, CC, and JMC contributed intellectually to the manuscript. DB conceived of and supervised this study and contributed to the manuscript. All authors read and approved the final manuscript.

**Table 2:** Environmental Conditions at Sites in Larderello, Tuscany and Pisciarelli

**Population**	**Micro-habitat**	**Sunlight**	**Temperature**	**pH**
Monte Rotondo-4 (MR4in)	Interlithic, Biomat among trachytic and detritic rocks, on the hot soil	Indirectly exposed	50°C	0–1
Monte Rotondo-5 (MR5en)	Endolithic, Biomat grew inside the thin sandy rock layers	Never exposed	37°C	1
Monte Rotondo-6 (MR6en)	Endolithic, Biomat grew inside the thin sandy rock layers	Never exposed	32°C	1
Sasso Pissano-1 (SP1in)	Interlithic, Biomat in a ravine of a crumbly wall between the detritus	Indirectly exposed	29–31°C	0.5–1
Sasso Pissano-3 (SP3en)	Endolithic, Biomat formed a thin layer inside a crumbly rock	Never exposed	30°C	1.0
Pisciarelli -B (PBen)	Endolithic, Biomat grew inside the layers of the alunite opal rock	Never exposed	18–30°C	0.5–1

**Table 3:** Genetic Differentiation Statistics Based on Population Parameters

Statistic	Estimate	Probability No differentiation *
Kst ^1^	0.021	0.0210
Kst* ^1^	0.015	0.0100
Zs ^1^	618.066	0.0230
Zs* ^1^	6.103	0.0100
Snn ^2^	0.675	0.0120

^1 ^[31].

^2 ^[32].

**Column headings**: *Probabilities obtained by the permutation test with 1000 replicates.
